# Individualized, dynamic, and full-course vancomycin dosing prediction: a study on the customized dose model

**DOI:** 10.3389/fphar.2024.1414347

**Published:** 2024-07-03

**Authors:** Xiangqing Song, Meizi Zeng, Tao Yang, Mi Han, Shipeng Yan

**Affiliations:** ^1^ Department of Pharmacy, Hunan Cancer Hospital/The Affiliated Cancer Hospital of Xiangya School of Medicine, Central South University, Changsha, China; ^2^ Office of Cancer Prevention Research, Hunan Cancer Hospital/The Affiliated Cancer Hospital of Xiangya School of Medicine, Central South University, Changsha, China

**Keywords:** vancomycin, mathematical modeling, therapeutic drug monitoring, pharmacokinetic/pharmacodynamic, dynamic administration, individual delivery

## Abstract

**Purpose:**

The single-point trough-based therapeutic drug monitoring (TDM) and Bayesian forecasting approaches are still limited in individualized and dynamic vancomycin delivery. Until recently, there has not yet been enough focus on the direct integration of pharmacokinetic/pharmacodynamic (PK/PD) and TDM to construct a customized dose model (CDM) for vancomycin to achieve individualized, dynamic, and full-course dose prediction from empirical to follow-up treatment. This study sought to establish CDM for vancomycin, test its performance and superiority in clinical efficacy prediction, formulate a CDM-driven full-course dosage prediction strategy to overcome the above challenge, and predict the empirical vancomycin dosages for six *Staphylococci* populations and four strains in patients with various creatinine clearance rates (CL_cr_).

**Methods:**

The PK/PD and concentration models derived from our earlier research were used to establish CDM. The receiver operating characteristic (ROC) curve, with the area under ROC curve (AUC_R_) as the primary endpoint, for 21 retrospective cases was applied to test the performance and superiority of CDM in clinical efficacy prediction by comparison to the current frequently-used dose model (FDM). A model with an AUC_R_ of at least 0.8 was considered acceptable. Based on the availability of TDM, the strategy of CDM-driven individualized, dynamic, and full-course dose prediction for vancomycin therapy was formulated. Based on the CDM, Monte Carlo simulation was used to predict the empirical vancomycin dosages for the target populations and bacteria.

**Results:**

Four CDMs and the strategy of CDM-driven individualized, dynamic, and full-course dose prediction for vancomycin therapy from empirical to follow-up treatment were constructed. Compared with FDM, CDM showed a greater AUC_R_ value (0.807 vs. 0.688) in clinical efficacy prediction. The empirical vancomycin dosages for six *Staphylococci* populations and four strains in patients with various CL_cr_ were predicted.

**Conclusion:**

CDM is a competitive individualized dose model. It compensates for the drawbacks of the existing TDM technology and Bayesian forecasting and offers a straightforward and useful supplemental approach for individualized and dynamic vancomycin delivery. Through mathematical modeling of the vancomycin dosage, this study achieved the goal of predicting doses individually, dynamically, and throughout, thus promoting “mathematical knowledge transfer and application” and also providing reference for quantitative and personalized research on similar drugs.

## 1 Introduction

As an antibiotic widely used for infections due to antibiotic-resistant Gram-positive bacteria, vancomycin (VAN) is often the last line of defense. Owing to high-concentration-related nephrotoxicity, low-concentration-related treatment failure, and sub-concentration-related bacterial resistance, how to personalize the administration of VAN to provide an appropriate therapeutic window has always been a topic worth discussing. To resolve this challenge, individualized dosing techniques based on therapeutic drug monitoring (TDM) and Bayesian forecasting (BF) are currently the two main methods (Rawson et al., 2021a).

Used as the current main technique for optimizing VAN doses, the prevailing TDM technique relies on tracking a single-point steady-state trough concentration (C_T-SS_) to establish the next dose needed to reach the trough target (typically 15–20 mg/L) or the ratio target (typically 400–600 mg·h/L) of the daily area under the concentration–time curve to minimal inhibitory concentration (AUC_24_/MIC) (Rybak, 2006). However, when TDM is implemented, dose adjustments are commonly made by rounding up and down (typically ±500 mg increments of per dose or ±4 h increments of dosing interval) in practice based on the measured C_T-SS_ value. Understandably, this truncated and all-in-one execution for dose optimization does not indicate truly individualized therapy since the value of ±500 mg increments 1) is not the actual value that needs to be adjusted but an approximate value that is easy to deliver, 2) may not apply to all patients due to the individual variations in physiopathology, and 3) fails to consider the essence of tailored dosage requirements that the dosage should be adjusted continuously and dynamically in response to the physiopathology or PK changes rather than making relatively fixed doses to account for various PK states. Single time-point C_T-SS_ is a useful clinical measurement; however, AUC_24_ is the primary PK/PD predictor of VAN activity. In many scenarios, single-point C_T-SS_ may not always correspond with the AUC_24_ (Neely et al., 2014), and single-point trough-only monitoring is insufficient to forecast the PK/PD characteristics of VAN according to the 2020 VAN guideline (Rybak et al., 2020). Although the TDM-based technique can predict the area under the concentration–time curve (AUC) and further guide AUC-based VAN dosage by using peak and trough concentrations (Pai et al., 2014; Heil et al., 2018), concurrent monitoring of them during the same disposition phase renders it mostly impracticable outside of a research environment. Consequently, the current single-point TDM technology and the customized dosages based on it do not fully personalize the administration of VAN unless real-time online monitoring technique is used.

As a sophisticated and advanced TDM technique, BF can estimate individual PK parameters and further determine individual doses to assure the accomplishment of PK/PD target based on individual data and population prior probability produced from the population model (Roberts et al., 2014; Avent and Rogers, 2019). BF has the ability of predicting the AUC and, thus, providing the AUC-guided dosage with the minimal PK sample (e.g., one or two concentrations). However, it requires specialized software or technical skill to be carried out, and the population PK model that is employed is frequently drawn from a limited target population and has not yet been widely validated (Rawson et al., 2021b). Presumably, there are still some limitations to its broad deployment in practice, especially in some small hospitals lacking technical support and financial input and in broad groups lacking a target population PK model. Currently, several open-access, online, BF-based VAN calculators, such as ClinCalc (https://clincalc.com/Vancomycin/) and VancoPK (https://www.vancopk.com/), provide free computing services for individualized AUC-based VAN dose recommendations. However, these calculators use a simplified AUC model that is derived from i.v. bolus (i.e., AUC_24_ = VAN daily dose/VAN clearance) to predict the VAN dose. This may cause a significant deviation between the predicted and desired dose due to the fact that VAN is often delivered via multiple intermittent infusion (MII) rather than via i.v. bolus, and the simplified AUC model is not well-suited for MII (Song and Wu, 2022). Therefore, the dose derived from these calculators is not necessarily a true personalized dose. Additionally, dose adjustments provided by these calculators are also made by rounding up and down (commonly ±250 mg increments of per dose) within a certain range of physiopathology or PK changes. Comparably, these calculators still have the same shortcomings in customized and dynamic dosing as the TDM-based dosing technique, although BF is used by them.

Consequently, in individualized and dynamic VAN dosing, these dosing technologies still have some limitations in application, and the recommended dosages based on them may still be unsatisfactory due to the abovementioned limitations although they provide useful clinical measurements or technical guidance. Therefore, an individualized dose-setting strategy for VAN that can generate continuous and dynamic (not cliff-like) dose recommendations based on ongoing physiopathology changes and is not dependent on specific software or technical supports is essential, thus prompting us to develop a customized dose model (CDM) for VAN to meet these requirements. Reportedly, individualized PK/PD optimization provides a possible gateway for tailored VAN dosage (Ambrose et al., 2007) and the pursuit of the desired strategy, which has piqued our interest in applying the PK/PD theory to individuate VAN dosing. Observationally, clinicians still prefer to utilize C_T-SS_ to determine subsequent dosage modification, although trough-only monitoring is no longer recommended in the 2020 VAN guideline (Rybak et al., 2020), thus explaining our tremendous appeal for employing a model with one even no C_T-SS_ to personalize VAN delivery. Given these ideas and the fact that little attention has been devoted to CDM and CDM-driven dosage prediction, by mathematical modeling of tailored VAN dosage, which integrates PK/PD and TDM (or C_T-SS_), we sought to create CDM for VAN to 1) achieve individualized, dynamic, and full-course VAN dosing prediction from empirical to follow-up therapy, regardless of whether C_T-SS_ is available; 2) construct a dose-tailored graph tool for empirical VAN treatment; and 3) offer a straightforward and practical approach for tailored and dynamic VAN dosing if successful. Through mathematical modeling of the VAN dosage, this study would achieve the goal of predicting individual doses. We believe that it would have a significant impact in promoting “mathematical knowledge transfer and application” and also provide reference for quantitative and personalized research on similar drugs.

## 2 Methods

### 2.1 Study design and setting

PK/PD and TDM (or C_T-SS_) were used to build the CDM based on the concentration models derived from our earlier research (Song and Wu, 2022); real-world data derived from 21 retrospective cases were applied to assess the superiority and performance of CDM in clinical efficacy prediction by comparison to the current frequently-used dose model (FDM), and based on the availability of TDM, the strategy of CDM-driven individualized, dynamic, and full-course dose prediction for VAN therapy was formulated. Based on CDM and using Monte Carlo simulation, VAN dosages in empirical therapy were predicted for six *Staphylococci* populations [i.e., *Staphylococcus aureus* (*S. aureus*), *Staphylococcus epidermidis* (*S*. *epidermidis*), methicillin-resistant *Staphylococcus aureus* (MRSA), *Staphylococcus* haemolyticus (*S. haemolyticus*), *Staphylococcus hominis* (*S. hominis*), and methicillin-susceptible *Staphylococcus aureus* (MSSA)] and for four strains with an MIC of 0.5, 1, 2, and 4 mg/L in patients with various creatinine clearance rates (CL_cr_), and these dosages were further used to create dose–CL_cr_ graphs.

### 2.2 Mathematical modeling of CDM

**FIGURE 1:**
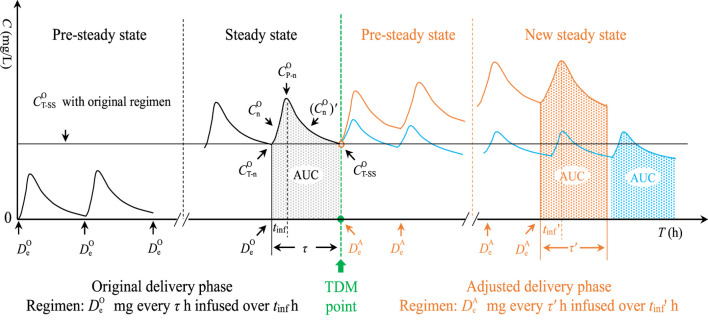
Concentration change in MII. MII, multiple intermittent infusion; $D_{e}^{O}$ , per dose in the original delivery phase; τ, dosing interval; t_inf_, infusion time; $C_{n}^{O}$ , concentration in the ascending branch of *n*th concentration curve in the original delivery phase; ${\left(C_{n}^{O}\right)}^{\prime }$ , concentration in the descending branch of the *n*th concentration curve in the original delivery phase; $C_{T-n}^{O}$ , trough concentration of the *n*th concentration curve in the original delivery phase; $C_{P-n}^{O}$ , peak concentration of the *n*th concentration curve in the original delivery phase; $C_{T-SS}^{O}$ , steady-state trough concentration in the original delivery phase; AUC, area under curve of per dose; $D_{e}^{A}$ , per dose in the adjusted delivery phase; τ′, adjusted dosing interval; t_inf_’, adjusted infusion time. The orange curve simulates that formed by increasing dosage according to $C_{T-SS}^{O}$ ; the blue curve simulates that formed by decreasing the dosage according to $C_{T-SS}^{O}$ .

In clinics, doctors frequently use MII to deliver VAN and determine the follow-up dose based on TDM results and the initial empirical dose. Concentration change of this dosing technique is outlined in Figure 1.

#### 2.2.1 Construction of CDM-1: an all-purpose steady-state CDM (when individualized parameters are available)

The following four formulas, derived from our previous study (Song and Wu, 2022), were used to create the CDM:

$$C_{n}^{O}=\frac{v\left(1-{e^{-K}}^{t_{\inf}}\right)}{CL_{\text{VAN}}}\cdot \frac{{e^{-K}}^{\left(\tau -t_{\inf}\right)}-{e^{-nK}}^{\left(\tau -t_{\inf}\right)}}{1-{e^{-K}}^{\left(\tau -t_{\inf}\right)}}+\frac{v\left(1-e^{-Kt}\right)}{CL_{\text{VAN}}},$$ (1)

$$C_{P-n}^{O}=\frac{v\left(1-{e^{-K}}^{t_{\inf}}\right)}{CL_{\text{VAN}}}\cdot \frac{1-{e^{-nK}}^{\left(\tau -t_{\inf}\right)}}{1-{e^{-K}}^{\left(\tau -t_{\inf}\right)}},$$ (2)

$${\left(C_{n}^{O}\right)}^{\prime }=\frac{v\left(1-{e^{-K}}^{t_{\inf}}\right)}{CL_{\text{VAN}}}\cdot \frac{1-{e^{-nK}}^{\left(\tau -t_{\inf}\right)}}{1-{e^{-K}}^{\left(\tau -t_{\inf}\right)}}\cdot {e^{-K}}^{t^{\prime }},$$ (3)

$$C_{T-n}^{O}=\frac{v\left(1-{e^{-K}}^{t_{\inf}}\right){e^{-K}}^{\left(\tau -t_{\inf}\right)}}{CL_{\text{VAN}}}\cdot \frac{1-{e^{-nK}}^{\left(\tau -t_{\inf}\right)}}{1-{e^{-K}}^{\left(\tau -t_{\inf}\right)}}.$$ (4)

Here, $C_{n}^{O}$ is the concentration in the ascending branch of the *n*th concentration curve in the original delivery phase, τ is the dosing interval, t_inf_ is the infusion time, v is the zero-order infusion rate calculated as per the dose in the original delivery phase ( $D_{e}^{O}$ ) divided by t_inf_, CL_VAN_ is the vancomycin elimination rate, K is the elimination rate constant, n is the *n*th concentration curve, e is the natural constant; $C_{P-n}^{O}$ is the peak concentration of the *n*th concentration curve in the original delivery phase, ${\left(C_{n}^{O}\right)}^{\prime }$ is the concentration in the descending branch of the *n*th concentration curve in the original delivery phase, and $C_{T-n}^{O}$ is the trough concentration of the *n*th concentration curve in the original delivery phase.

According to Figure 1, the trapezoidal-based AUC in any curve can be expressed as follows:

$$\text{AUC}=\int_{0}^{t_{\inf}}C_{n}^{O}dt+\int_{0}^{\tau -t_{\inf}}{\left(C_{n}^{O}\right)}^{\prime }dt.$$ (5)

Equation 1 and Equation 3 are integrated into Equation 5. Then,

$$\text{AUC}=\int_{0}^{t_{\inf}}\left[\frac{v\left(1-{e^{-K}}^{t_{\inf}}\right)}{CL_{\text{VAN}}}\cdot \frac{{e^{-K}}^{\left(\tau -t_{\inf}\right)}-{e^{-nK}}^{\left(\tau -t_{\inf}\right)}}{1-{e^{-K}}^{\left(\tau -t_{\inf}\right)}}+\frac{v\left(1-e^{-Kt}\right)}{CL_{\text{VAN}}}\right]dt+\int_{0}^{\tau -t_{\inf}}\frac{v\left(1-{e^{-K}}^{t_{\inf}}\right)}{CL_{\text{VAN}}}\cdot \frac{1-{e^{-nK}}^{\left(\tau -t_{\inf}\right)}}{1-{e^{-K}}^{\left(\tau -t_{\inf}\right)}}\cdot {e^{-K}}^{t}dt.$$ (6)

The definite integral is converted, and the expression of v = $D_{e}^{O}$ /t_inf_ is integrated. Then,

$$\text{AUC}=\frac{D_{e}^{O}\left(1-e^{-Kt_{\inf}}\right)}{CL_{\text{VAN}}}\left[\frac{{e^{-K}}^{\left(\tau -t_{\inf}\right)}-{e^{-nK}}^{\left(\tau -t_{\inf}\right)}}{1-{e^{-K}}^{\left(\tau -t_{\inf}\right)}}+\frac{1}{1-e^{-Kt_{\inf}}}-\frac{{e^{-nK}}^{\left(\tau -t_{\inf}\right)}}{t_{\inf}K}\right].$$ (7)

Since 24/τ curves are formed in 24 h, then,

$${\text{AUC}}_{24}=\frac{24D_{e}^{O}\left(1-e^{-Kt_{\inf}}\right)}{\tau CL_{\text{VAN}}}\left[\frac{{e^{-K}}^{\left(\tau -t_{\inf}\right)}-{e^{-nK}}^{\left(\tau -t_{\inf}\right)}}{1-{e^{-K}}^{\left(\tau -t_{\inf}\right)}}+\frac{1}{1-e^{-Kt_{\inf}}}-\frac{{e^{-nK}}^{\left(\tau -t_{\inf}\right)}}{t_{\inf}K}\right].$$ (8)

Hence,

$$\frac{{\text{AUC}}_{24}}{\text{MIC}}=\frac{24D_{e}^{O}\left(1-e^{-Kt_{\inf}}\right)}{\tau CL_{\text{VAN}}\text{MIC}}\left[\frac{{e^{-K}}^{\left(\tau -t_{\inf}\right)}-{e^{-nK}}^{\left(\tau -t_{\inf}\right)}}{1-{e^{-K}}^{\left(\tau -t_{\inf}\right)}}+\frac{1}{1-e^{-Kt_{\inf}}}-\frac{{e^{-nK}}^{\left(\tau -t_{\inf}\right)}}{t_{\inf}K}\right].$$ (9)

Due to the daily dose in the original delivery phase (defined as $D_{d}^{O}$ ) = $D_{e}^{O}$ ×(24/τ), then,

$$\frac{{\text{AUC}}_{24}}{\text{MIC}}=\frac{D_{d}^{O}\left(1-e^{-Kt_{\inf}}\right)}{CL_{\text{VAN}}\text{MIC}}\left[\frac{{e^{-K}}^{\left(\tau -t_{\inf}\right)}-{e^{-nK}}^{\left(\tau -t_{\inf}\right)}}{1-{e^{-K}}^{\left(\tau -t_{\inf}\right)}}+\frac{1}{1-e^{-Kt_{\inf}}}-\frac{{e^{-nK}}^{\left(\tau -t_{\inf}\right)}}{t_{\inf}K}\right].$$ (10)

Since the AUC_24_/MIC target of 400 (regarded as a target constant “φ”) should be achieved in optimal VAN therapy (Rybak et al., 2020), an optimal daily dose (defined as $D_{d}^{A}$ ) should be delivered. Then,

$$\varphi =\frac{D_{d}^{A}\left(1-e^{-Kt_{\inf}}\right)}{CL_{\text{VAN}}\text{MIC}}\left[\frac{{e^{-K}}^{\left(\tau -t_{\inf}\right)}-{e^{-nK}}^{\left(\tau -t_{\inf}\right)}}{1-{e^{-K}}^{\left(\tau -t_{\inf}\right)}}+\frac{1}{1-e^{-Kt_{\inf}}}-\frac{{e^{-nK}}^{\left(\tau -t_{\inf}\right)}}{t_{\inf}K}\right].$$ (11)

By transformation,

$$D_{d}^{A}=\frac{\varphi CL_{\text{VAN}}\text{MIC}}{\left(1-e^{-Kt_{\inf}}\right)\left[\frac{{e^{-K}}^{\left(\tau -t_{\inf}\right)}-{e^{-nK}}^{\left(\tau -t_{\inf}\right)}}{1-{e^{-K}}^{\left(\tau -t_{\inf}\right)}}+\frac{1}{1-e^{-Kt_{\inf}}}-\frac{{e^{-nK}}^{\left(\tau -t_{\inf}\right)}}{t_{\inf}K}\right]}.$$ (12)

When n reaches infinity or the VAN level achieves a stable state, e^-nK(τ-t_inf_)^ approaches zero. Then,

$$\left(i.e.,\text{CDM}-1\right):D_{d}^{A}=\frac{\varphi CL_{\text{VAN}}\text{MIC}\left({e^{K}}^{\tau }-{e^{K}}^{t_{\inf}}\right)}{e^{K\tau }-1}.$$ (13)

CDM-1 reveals a universal steady-state dose model. The following are three dependent CDMs based on CDM-1 and the number of concentrations available.

#### 2.2.2 Construction of CDM-2: a full-population-based steady-state CDM (when no C_T-ss_ is available)

As an alternative to estimating individual *CL*_VAN_ and *K*, the following population models of *CL*_VAN_ and *K* for VAN established by Matzke et al. (1984) were employed:

$$CL_{\text{VAN}}\left(\text{ml}/\min\right)=0.689\times CL_{\text{cr}}\;\left(\text{ml}/\min\right)+3.66,$$ (14)

$$K\;\left(h^{-1}\right)=0.00083\times CL_{\text{cr}}\;\left(\text{ml}/\min\right)+0.0044.$$ (15)

Incorporating Equation 14 and Equation 15 into Equation 13, then,

$$\left(i.e.,\text{CDM}-2\right):D_{d}^{A}=\frac{\varphi \left(0.689CL_{cr}+3.66\right)\text{MIC}\left[e^{\left(0.00083CL_{cr}+0.0044\right)\tau }-e^{\left(0.00083CL_{cr}+0.0044\right)t_{\inf}}\right]}{e^{\left(0.00083CL_{cr}+0.0044\right)\tau }-1}.$$ (16)

#### 2.2.3 Construction of CDM-3: a semi-population-based steady-state CDM (when one C_T-ss_ is available)

In Equation 4, when n reaches infinity or the VAN level achieves a steady state, e^-nK(τ-t_inf_)^ and $C_{T-n}^{O}$ approach zero and $C_{T-SS}^{O}$ , respectively. Then,

$$C_{T-SS}^{O}=\frac{v\left(1-{e^{-K}}^{t_{\inf}}\right){e^{-K}}^{\left(\tau -t_{\inf}\right)}}{CL_{\text{VAN}}}\cdot \frac{1}{1-{e^{-K}}^{\left(\tau -t_{\inf}\right)}}.$$ (17)

Multiplying Equation 13 by Equation 17, then,

$$D_{d}^{A}=\frac{v\varphi \text{MIC}}{C_{T-SS}^{O}}\cdot \frac{e^{Kt_{\inf}}-1}{{e^{K}}^{\tau }-1}.$$ (18)

Because $D_{d}^{O}$ = $D_{e}^{O}$ ×(24/τ) and v = $D_{e}^{O}$ /t_inf_, then,

$$D_{d}^{A}=D_{d}^{O}\cdot \frac{\varphi \text{MIC}}{24C_{T-SS}^{O}}\cdot \frac{\tau }{t_{\inf}}\cdot \frac{e^{Kt_{\inf}}-1}{{e^{K}}^{\tau }-1}.$$ (19)

Integrating Eq. 15 into Eq. 19, then,

$$\left(i.e.,\text{CDM}-3\right):D_{d}^{A}=D_{d}^{O}\cdot \frac{\varphi \text{MIC}}{24C_{T-SS}^{O}}\cdot \frac{\tau }{t_{\inf}}\cdot \frac{e^{\left(0.00083CL_{\text{cr}}+0.0044\right)t_{\inf}}-1}{{e^{\left(0.00083CL_{\text{cr}}+0.0044\right)}}^{\tau }-1}.$$ (20)

#### 2.2.4 Construction of CDM-4: a non-population-based steady-state CDM [when concomitant C_T-ss_ and C_P-ss_ (steady-state peak) during the same disposition phase are available]

In Equation 2 and Equation 4, when n reaches infinity or the VAN level achieves the steady state, e^-nK(τ-t_inf_)^, $C_{T-n}^{O}$ , and $C_{P-n}^{O}$ approach zero, $C_{T-SS}^{O}$ , and $C_{P-SS}^{O}$ , respectively. Then,

$$C_{P-SS}^{O}=\frac{v\left(1-{e^{-K}}^{t_{\inf}}\right)}{CL_{\text{VAN}}}\cdot \frac{1}{1-{e^{-K}}^{\left(\tau -t_{\inf}\right)}},$$ (21)

$$C_{T-SS}^{O}=\frac{v\left(1-{e^{-K}}^{t_{\inf}}\right){e^{-K}}^{\left(\tau -t_{\inf}\right)}}{CL_{\text{VAN}}}\cdot \frac{1}{1-{e^{-K}}^{\left(\tau -t_{\inf}\right)}}.$$ (22)

Merging Equation 22 and Equation 23, then,

$$K=\frac{1}{\tau -t_{\inf}}\cdot \mathrm{Ln}\frac{C_{P-SS}^{O}}{C_{T-SS}^{O}}.$$ (23)

Incorporating Equation 23 into Equation 21, then,

$$CL_{\text{VAN}}=\frac{v\cdot \left[1-{\left(\frac{C_{T-SS}^{O}}{C_{P-SS}^{O}}\right)}^{\frac{t_{\inf}}{\tau -t_{\inf}}}\right]\cdot \ln\frac{C_{P-SS}^{O}}{C_{T-SS}^{O}}}{C_{P-SS}^{O}\cdot \left(\ln\frac{C_{P-SS}^{O}}{C_{T-SS}^{O}}-1\right)}.$$ (24)

Integrating Equation 23 and Equation 24 into Equation 13, then,

$$D_{d}^{A}=\frac{v\varphi \text{MIC}\left[1-{\left(\frac{C_{T-SS}^{O}}{C_{P-SS}^{O}}\right)}^{\frac{t_{\inf}}{\tau -t_{\inf}}}\right]\left[{\left(\frac{C_{P-SS}^{O}}{C_{T-SS}^{O}}\right)}^{\frac{\tau }{\tau -t_{\inf}}}-{\left(\frac{C_{P-SS}^{O}}{C_{T-SS}^{O}}\right)}^{\frac{t_{\inf}}{\tau -t_{\inf}}}\right]\ln\frac{C_{P-SS}^{O}}{C_{T-SS}^{O}}}{C_{P-SS}^{O}\cdot \left(\ln\frac{C_{P-SS}^{O}}{C_{T-SS}^{O}}-1\right)\left[{\left(\frac{C_{P-SS}^{O}}{C_{T-SS}^{O}}\right)}^{\frac{\tau }{\tau -t_{\inf}}}-1\right]},$$ (25)

Due to $D_{d}^{O}=D_{e}^{O}\times (24/\tau )$ and $v=D_{e}^{O}/t_{inf}$ , then,

$$\left(i.e.,\text{CDM}-4\right):D_{d}^{A}=D_{d}^{O}\cdot \frac{\varphi \text{MIC}}{24C_{P-SS}^{O}}\cdot \frac{\tau }{t_{\inf}}\cdot \frac{\ln\frac{C_{P-SS}^{O}}{C_{T-SS}^{O}}\cdot \left[1-{\left(\frac{C_{T-SS}^{O}}{C_{P-SS}^{O}}\right)}^{\frac{t_{\inf}}{\tau -t_{\inf}}}\right]\left[{\left(\frac{C_{P-SS}^{O}}{C_{T-SS}^{O}}\right)}^{\frac{\tau }{\tau -t_{\inf}}}-{\left(\frac{C_{P-SS}^{O}}{C_{T-SS}^{O}}\right)}^{\frac{t_{\inf}}{\tau -t_{\inf}}}\right]}{\left(\ln\frac{C_{P-SS}^{O}}{C_{T-SS}^{O}}-1\right)\left[{\left(\frac{C_{P-SS}^{O}}{C_{T-SS}^{O}}\right)}^{\frac{\tau }{\tau -t_{\inf}}}-1\right]}.$$ (26)

### 2.3 Evaluation of CDM

#### 2.3.1 Real-world data collection and literature review

Real-world data taken from publications were employed to assess the CDM. Case reports were preferred since they frequently included a detailed explanation of the changes made to TDM, dose modification, and efficacy, making it easier to analyze their relationships. The mainstream English and Chinese databases, including PubMed (https://pubmed.ncbi.nlm.nih.gov/), CNKI (https://www.cnki.net/), CBMdisc (http://www.sinomed.ac.cn/index.jsp), China Online Journals (https://www.wanfangdata.com.cn/index.html), and VIP Journal Service Platform (http://qikan.cqvip.com/), were systematically searched for TDM of VAN published up to 1 October 2023. The keywords (vancomycin) AND (pharmacokinetic OR pharmacokinetics) AND (therapeutic drug monitoring OR TDM OR concentration) were used in the search strategy. Additional publications cited in the references of the identified publications were also screened. The publications that met the following inclusion and exclusion criteria were included: 1) inclusion criteria: cases without language and gender restrictions but with at least one VAN trough-monitoring; 2) exclusion criteria: cases with renal replacement therapy, morbid obesity (i.e., body mass index >40 kg/m^2^ or body weight ≥120 kg), <16 years, VAN therapy using non-intermittent infusion, or insufficient data on efficacy. Collected data included sex, age, body weight, serum creatinine (*S*_cr_) or *CL*_cr_ before and after (if measured) VAN therapy, MIC (if available), original and amended VAN regimen, TDM results, and clinical efficacy.

#### 2.3.2 Superiority and performance testing of the CDM

Using the real-world data collected, the superiority and performance of the CDM in clinical efficacy prediction were evaluated by comparing with FDM (i.e., $D_{d}^{A}$ or $D_{d}^{O}$ = φ·MIC·CL_van_, which was generated from the model of CL_van_ = daily dose/AUC_24_ (Guo et al., 2019a; vancomycin calculator, n.d.; vancomycin dosing calculator, n.d.)). As AUC_24_ is a quantifiable primary consensus index for VAN efficacy judgment and dosage formulation (Rybak et al., 2020), we equivalently converted CDM into an AUC_24_ model (see the note of Table 2) and used the comparison of AUC_24_ of VAN regimens in the collected cases to inversely corroborate the superiority and performance of the CDM. AUC_24_ of VAN regimens was calculated based on CDM and FDM. The Bland–Altman plot of AUC_24_ was first employed to evaluate the consistency of CDM and FDM (Guo et al., 2019a). CDM and FDM were considered consistent in predicting the dose if the AUC_24_ bias was within ±48 mg·h/L (a maximum permitted estimate obtained by multiplying 24 h by an acceptable concentration bias of ±2 mg/L (Gastmans et al., 2022)). Otherwise, additional index for 1) area under receiver operating characteristic (ROC) curve (AUC_R_) of AUC_24_ on clinical efficacy and 2) correlations and consistency of the predicted efficacy on clinical efficacy were measured. AUC_R_, as the primary endpoint, and correlations and consistency, as the secondary endpoint, were used to evaluate the superiority and performance of CDM in clinical efficacy prediction. An alpha of 0.05 was used as the cut-off for significance. Efficacy was classified into “valid” and “invalid” by a binary classification. Predicted efficacy of VAN regimens was defined as “valid” if the predicted AUC_24_ is ≥ 400 or “invalid” if the predicted AUC_24_ is < 400. Clinical or actual efficacy of VAN regimens was extracted from the cases. A dose model with an AUC_R_ of ≥0.80 was considered acceptable (Nahm, 2022), and together with a higher AUC_R_, correlation, and consistency, it was considered optimal. Statistical analysis and plotting were conducted by OriginPro 2019b.

### 2.4 Strategy formulation of individualized, dynamic, and full-course VAN dosing prediction from empirical to follow-up therapy

Based on the availability and variability of the parameters in CDMs throughout VAN treatment, we selected appropriate CDMs to calculate empirical or adjusted individual daily doses and further determined specific dosing regimens according to the calculated daily dosage, τ, and t_inf_.

### 2.5 Required $D_{d}^{O}$ and construction of $D_{d}^{O}$ –CL_cr_ graphs for empirical VAN therapy

The required empirical $D_{d}^{O}$ for the top six *Staphylococci* populations (including *S. aureus*, *S. epidermidis*, MRSA, *S. haemolyticus*, *S. hominis*, and MSSA) and for four strains with a MIC of 0.5, 1, 2, and 4 mg/L in patients with various CL_cr_ was predicted based on Monte Carlo simulations and CDM-2 and further used to construct $D_{d}^{O}$ –CL_cr_ graphs. In Monte Carlo simulations, custom distributions for MIC of strains or MIC frequency of population; τ of 6, 8, and 12 h; φ of 400 and CL_cr_ ranging from 10 to 300 mL/min with a 10 mL/min increment; uniform distributions for t_inf_ of commonly 1–3 h (an estimated time determined by per dose of typically 0.5–2 g (Filippone et al., 2017); and infusion rate of allowable 10–15 mg/min (Rybak et al., 2020) in MII) were assumed. The top six *Staphylococci* populations and their MIC frequency were derived from the European Committee on Antimicrobial Susceptibility Testing (https://mic.eucast.org/). A 95% confidence interval was set. The mean $D_{d}^{O}$ at a φ of 400 for 5,000-subject simulations was considered sufficient. Monte Carlo simulations were conducted by Oracle Crystal Ball 11.1.2.

## 3 Results

### 3.1 Mathematical modeling of CDM

A universal CDM (i.e., CDM-1) and three deuterogenic CDMs (i.e., CDM-2, 3, and 4), including two concentration-independent CDMs (i.e., CDM-1 and 2) and two concentration-dependent CDMs (i.e., CDM-3 and 4), were established. CDM-1 allowed for both precise (if individual parameters are obtained and integrated) and rough (if population parameters are obtained and integrated) dose predictions. Meanwhile, it revealed the fact that at the same PK/PD exposure, the daily dose can be decreased by extending t_inf_ and/or shortening τ. This finding is of great significance for optimizing the dose. CDM-2 revealed the relationship for determining $D_{d}^{O}$ based on the individual parameters and PK/PD target, while CDM-3 and CDM-4 provided information on how to compute $D_{d}^{A}$ based on $D_{d}^{O}$ and its C_T-SS_ and PK/PD target. CDM-2, 3, and 4 indicated that $D_{d}^{A}$ would be constant at a steady CL_cr_ and C_T-SS_, implying that a fixed dosing regimen can be continuously used for patients with stable hepatorenal function, given that CL_cr_ and C_T-SS_ in these patients often remain constant. Understandably, patients with unstable hepatorenal function should have a dynamic dosing regimen since their CL_cr_ and C_T-SS_ typically fluctuate. Additionally, CMD-based dose calculation can be performed by the custom function editing of Microsoft^®^ Excel^®^ 2019 MSO (version 2112) or the elementary arithmetic editing of an application (e.g., Casio 991ES calculator) or a calculator (e.g., Casio fx-991ES) with advanced computing functions, simplifying the dose formulation process because it does not need professional software or technical support.

### 3.2 Evaluation of CDM

#### 3.2.1 Real-world data collection

Table 1 displays the collected data on 21 retrospective cases. The patients had various renal functions, and all received VAN therapy via MII to treat various Gram-positive bacterial infections involving the lung, knee prosthesis implantation, sepsis, bloodstream, encephalopyosis, and abdomen. All were sampled for trough monitoring. All but cases 16, 19, and 20 had samples taken for bacterial cultures; only 7 of 21 had MIC reports. Clinical efficacy of 43 regimens in all (including 21 initial regimens and 22 modified regimens) was assessed and extracted.

**TABLE 1:** Clinical data on the collected cases.

References	No.	Physiological information					Infection information				VAN therapy and clinical outcome						
		Sex	Age (year)	Body weight (kg)	S_cr_ ^a^ (μmol/L)	Calculated *CL*_cr_ ^b^ (mL/min)	Infected type	Specimen	Infectious strain^c^	MIC (mg/L)	Dosing regimen (h)	S_cr_ ^d^ (μmol/L)	Calculated *CL*_cr_ ^e^ (mL/min)	TDM on which day (d)	*C*O T (mg/L)	Duration days of the regimen (d)	Clinical outcome in references^f^
Liu et al. (2021)	1	M	57	62.5	65.1	97.4	Pulmonary infection	Sputum	MRSA	-	1 g q 12	81.8	77.5	3	8.2	3	Progressive
											1 g q 8	75.5	84.0	5	13.9	5	Effectual
	2	F	68	45	35.3	95.4	Knee prosthesis implantation infection	Joint fluid	*S. aureus*	-	1 g q 12	35.0	96.2	2	23.5	2	Effectual
											0.5 g q 8	33.1	101.7	6	15.2	9	Restorative
Zhang et al. (2015)	3	M	50	-	34.0	194.2^g^	Bloodstream infection	Blood	*S. schleiferi*	2	0.5 g q 8	-	-	22	5.69	22	Progressive
											1 g q 8	110.0	60.0	35	11.25	35	Restorative
	4	M	55	-	50.0	135.1^g^	Bloodstream infection	Blood	*S. hyicus*	2	1 g q 12	-	-	4	4.29	4	Progressive
											1 g q 8	53.5	126.3	9	15.76	9	Restorative
	5	M	36	-	63.9	119.4^g^	Bloodstream infection	Blood	*S. epidermidis*	2	1 g q 12	-	-	8	4.35	8	Ineffective
											1 g q 8	52.3	145.9	13	5.16	16	Ineffective
	6	F	49	-	55.6	85.0^g^	Bloodstream infection	Blood	*S. hominis*	2	1 g q 12	-	-	30	2.82	30	Ineffective
											1 g q 8	30.4	155.5	34	4.54	39	Ineffective
	7	F	24	-	38.9	155.0^g^	Bloodstream infection	Blood	*S. hominis*	1	1 g q 12	-	-	3	2.07	3	Progressive
											1 g q 8	32.0	188.4	12	6.92	23	Progressive
Qu et al. (2022)	8	M	17	65	44.4	220.1	Septic shock	Blood	MRSA	0.5	1 g q 12	-	220.0^g^	3	8.43	3	Progressive
											1 g q 8	-	-	4	5.64	8	Restorative
Shi et al. (2017)	9	F	53	62	-	103.0^g^	Wound and bloodstream infection	Wounds and blood	MRSA	-	1 g q 8	64.0	103.0	1	21.9	3	Effectual
											1 g q 12	-	-	5	14.7	9	Restorative
Chen et al. (2023)	10	M	50	74	56.3	145.2^g^	Empirical therapy	Blood	MRSE	-	1 g q 12	-	-	3	3.9	3	Ineffective
											1 g q 8	45.1	181.3^g^	2	7.5	9	Ineffective
Wu et al. (2023)	11	M	58	73	-	107.6^g^	Encephalopyosis	Sanies	MRCNS	-	1 g q 12	-	113.7^g^	5	6.6	8	Progressive
											1 g q 8	-	105.3^g^	6	15.2	17	Progressive
Liu et al. (2022)	12	M	49	85	-	131.2^g^	Abdominal infection followed by bloodstream infection	Blood	*E. faecium*	-	1 g q 12	57.2	131.2^g^	6	7.65	6	Ineffective
											1 g q 8	47.2	159.0	2	16.9	17	Restorative
Zhang et al. (2021)	13	M	51	-	48.0	204.9^g^	Sepsis	Blood	*S. aureus*	1	1 g q 12	33.0	298.0^g^	3	6.2	3	Progressive
											1 g q 8	37.0	265.8^g^	2	8.3	2	Progressive
											1.25 g q 8	36.0	273.2^g^	5	7.0	5	Progressive
											1 g q 6	45.0	218.5^g^	4	11.8	4	Progressive
Yang et al. (2019)	14	M	20	90	74.0	126.2^g^	Septic shock	Blood	MRS. hominis	-	1 g q 12	68.0	130.7^g^	4	3.3	9	Ineffective
											1 g q 8	118.0	76.1^g^	5	25.5	13	Progressive
											1 g q 12	57.0	141.5^g^	-	-	7	Effectual
Huang et al. (2016)	15	M	67	60	53.7	99.7	Joint prosthesis implantation infection	Joint fluid	*S. epidermidis*	-	1 g q 12	48.8	109.7	3	25.2	3	Effectual
											0.5 g q 8	50.6	105.8	3	17.6	3	Effectual
	16	M	66	65	47.9	122.8	Septic shock	-	Suspicious *S. aureus*	-	1 g q 12	187.3	31.4	3	28.2	3	Effectual
											0.5 g q 8	136.9	43.0	3	18.9	3	Effectual
	17	F	58	43	53.7	68.2	Sepsis	Blood	*S. aureus*	-	1 g q 12	-	-	3	8.8	3	Progressive
											1 g q 8	50.4	72.7	3	19.9	3	Effectual
Lai et al. (2019)	18	M	50	62	85.7	79.9^g^	Septicemia	Blood	MRSA	-	1 g q 12	175.2	39.1^g^	3	19.7	3	Effectual
											1 g q 24	130.6	45.1^g^	3	12.75	3	Effectual
Lai et al. (2018)	19	F	74	45	236.0	13.1	Sepsis	-	Suspicious *S. aureus*	-	0.5 g q 24	127.8	24.1	3	18.14	3	Effectual
	20	M	42	60	161.0	44.6	Septic shock	-	Suspicious *S. aureus*	-	0.5 g q 12	49.5	145.2	2	7.67	2	Progressive
											1 g q 12	34.8	206.6	2	15.6	7	Effectual
	21	M	70	58	111.0	44.7	Pulmonary infection	Sputum	MRSA	-	0.5 g q 6	51.6	96.2	5	18.2	5	Effectual

^a^ S_cr_ before VAN therapy.

^b^ *CL*_cr_ before VAN therapy.

^c^ MRSA, methicillin-resistant *Staphylococcus aureus*; *S. aureus*, *Staphylococcus aureus*; *S. schleiferi*, *Staphylococcus schleiferi*; *S. hyicus*, *Staphylococcus hyicus*; *S. epidermidis*, *Staphylococcus epidermidis*; *S. hominis*, *Staphylococcus hominis*; MRSE, methicillin-resistant *Staphylococcus epidermidis*; MRCNS, methicillin-resistant coagulase negative *Staphylococci*; *E. faecium*, *Enterococcus faecium*; MRS., hominis, methicillin-resistant *Staphylococcus hominis*.

^d^ S_cr_ after VAN therapy.

^e^ *CL*_cr_ after VAN therapy.

^f^ Four levels for efficacy evaluation were classified: 1) restorative: clinical symptoms, signs, and laboratory and etiological examination all returned to normal; 2) effectual: the illness state was considerably improved, but one of the aforementioned indicators did not fully recover; 3) progressive: the illness state was improved, but the symptoms or signs partially disappeared or improved; 4) ineffective: the illness state was not significantly improved or aggravated. In efficacy evaluation, even in cases where different bacteria and antibiotics were combined, only the efficacy of VAN was assessed.

^g^ Directly provided by the literature. “-”, not available.

#### 3.2.2 Superiority and performance testing of CDM

**FIGURE 2:**
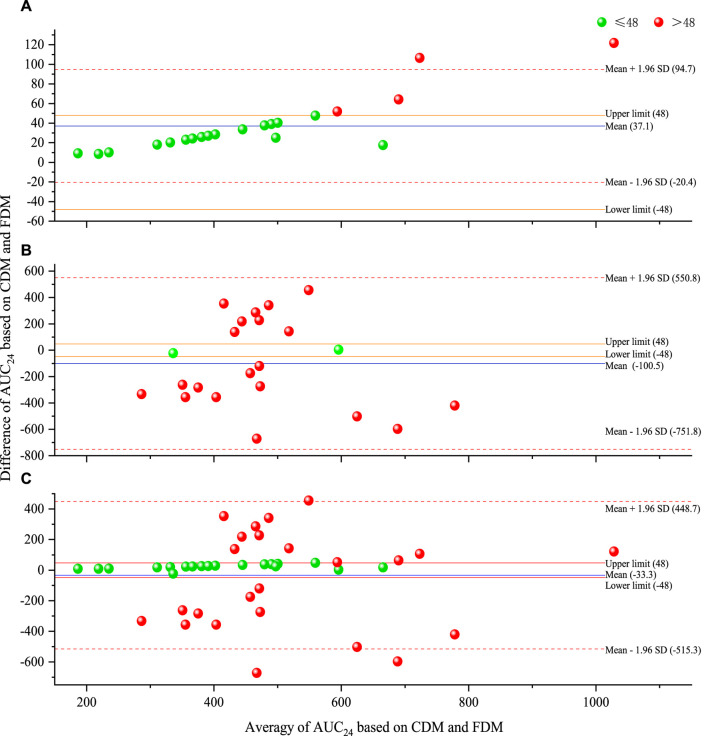
Bland–Altman plot of predicted AUC_24_ based on CDM and FDM [**(A)** for original regimens (N = 21), **(B)** for adjusted regimens (N = 22), and **(C)** for all regimens (N = 43)]. AUC_24_, daily area under the concentration–time curve to minimal inhibitory concentration; CDM, customized dose models; FDM, frequently used dose model; SD, standard deviation.

Table 2 presents the predicted AUC_24_ and efficacy of 43 regimens (including 21 original regimens and 22 adjusted regimens) in 21 cases. Figure 2 shows a Bland–Altman plot comparing the predicted AUC_24_ based on CDM and FDM. Compared with FDM, CDM showed an average increase of 37.1 mg·h/L in the original regimens, a decrease of 100.5 mg·h/L in the adjusted regimens, and a decrease of 33.3 mg·h/L in the total regimens in terms of AUC_24_. A total of 19.0% (4 of 21) of points in 21 original regimens, 90.9% (20 of 22) of points in 22 adjusted regimens, and 55.8% (24 of 43) of points in 43 total regimens were beyond the limit of ±48 mg·h/L, indicating that CDM and FDM were inconsistent in predicting AUC_24_ and the resulting dose, especially for adjusted regimens.

**TABLE 2:** Predicted AUC_24_, predicted efficacy, and clinical efficacy.

No.	Infectious strain^a^	Dosing regimen (h)	Daily dose(mg)	τ (h)	t_inf_ (h)	Predicted AUC_24_		Predicted efficacy^b^		Clinical efficacy
						CDM^c^	FDM^d^	CDM	FDM	
1	MRSA	1 g q 12	2,000	12	1.5	510.1	471.0	1	1	0
		1 g q 8	3,000	8	1.5	373.6	876.3	0	1	1
2	*S. aureus*	1 g q 12	2,000	12	1.5	520.7	480.4	1	1	1
		0.5 g q 8	1,500	8	0.75	584.9	357.4	1	0	1
3	*S. schleiferi*	0.5 g q 8	1,500	8	0.75	191.1	181.9	0	0	0
		1 g q 8	3,000	8	1.5	501.4	363.8	1	0	1
4	*S. hyicus*	1 g q 12	2,000	12	1.5	367.6	344.6	0	0	0
		1 g q 8	3,000	8	1.5	233.5	516.9	0	1	1
5	*S. epidermidis*	1 g q 12	2,000	12	1.5	416.4	388.0	1	0	0
		1 g q 8	3,000	8	1.5	225.3	582.0	0	1	0
6	*S. hominis*	1 g q 12	2,000	12	1.5	583.0	535.5	1	1	0
		1 g q 8	3,000	8	1.5	131.4	803.2	0	1	0
7	*S. hominis*	1 g q 12	2,000	12	1.5	319.9	301.9	0	0	0
		1 g q 8	3,000	8	1.5	120.1	452.9	0	1	0
8	MRSA	1 g q 12	2,000	12	1.5	223.2	214.6	0	0	0
		1 g q 8	3,000	8	1.5	608.7	322.1	1	0	1
9	MRSA	1 g q 8	3,000	8	1.5	776.5	670.0	1	1	1
		1 g q 12	2,000	12	1.5	588.9	446.7	1	1	1
10	MRSE	1 g q 12	2,000	12	1.5	341.7	321.4	0	0	0
		1 g q 8	3,000	8	1.5	219.3	482.1	0	1	0
11	MRCNS	1 g q 12	2,000	12	1.5	461.9	428.4	1	1	0
		1 g q 8	3,000	8	1.5	335.8	609.8	0	1	0
12	*E. faecium*	1 g q 12	2,000	12	1.5	378.6	354.4	0	0	0
		1 g q 8	3,000	8	1.5	411.3	531.6	1	1	1
13	*S. aureus*	1 g q 12	2,000	12	1.5	240.3	230.2	0	0	0
		1 g q 8	3,000	8	1.5	592.2	239.3	1	0	0
		1.25 g q 8	3,750	8	2	553.1	334.6	1	0	0
		1 g q 6	4,000	6	1.5	324.6	347.4	0	0	0
14	MRS. hominis	1 g q 12	2,000	12	1.5	393.8	367.9	0	0	0
		1 g q 8	3,000	8	1.5	177.1	533.5	0	1	0
		1 g q 12	2,000	12	1.5	597.5	594.3	1	1	1
15	*S. epidermidis*	1 g q 12	2,000	12	1.5	498.4	460.7	1	1	1
		0.5 g q 8	1,500	8	0.75	656.7	315.5	1	0	1
16	*S. aureus*	1 g q 12	2,000	12	1.5	404.7	377.6	1	0	1
		0.5 g q 8	1,500	8	0.75	568.3	988.4	1	1	1
17	*S. aureus*	1 g q 12	2,000	12	1.5	722.2	658.1	1	1	0
		1 g q 8	3,000	8	1.5	390.0	987.2	0	1	1
18	MRSA	1 g q 12	2,000	12	1.5	619.6	567.8	1	1	1
		1 g q 24	1,000	24	1.5	369.6	544.7	0	1	1
19	*S. aureus*	0.5 g q 24	500	24	0.75	674.4	656.9	1	1	1
20	*S. aureus*	0.5 g q 12	1,000	12	0.75	509.6	484.6	1	1	0
		1 g q 12	2,000	12	1.5	776.5	321.4	1	0	1
21	MRSA	0.5 g q 6	2,000	6	0.75	1,089.1	967.4	1	1	1

^a^ MRSA, methicillin-resistant *Staphylococcus aureus*; *S. aureus*, *Staphylococcus aureus*; *S. schleiferi*, *Staphylococcus schleiferi*; *S. hyicus*, *Staphylococcus hyicus*; *S. epidermidis*, *Staphylococcus epidermidis*; *S. hominis*, *Staphylococcus hominis*; MRSE, methicillin-resistant *Staphylococcus epidermidis*; MRCNS, methicillin-resistant coagulase-negative *Staphylococci*; *E. faecium*, *Enterococcus faecium*; MRS. hominis, methicillin-resistant *Staphylococcus hominis*.

^b^ In the binary classification for efficacy, the terms “restorative” and “effectual” in four-level efficacy evaluation were classified as “valid,” whereas “progressive” and “ineffective” were classified as “invalid.”

^c^ Determined by the following models.

For original regimens (derived from CDM-2): $AUC_{24}=\frac{D_{d}^{A}\left[e^{\left(0.00083CL_{\text{cr}}+0.0044\right)\tau }-1\right]}{\left(0.689CL_{\text{cr}}+\;3.66\right)\left[e^{\left(0.00083CL_{\text{cr}}+\;0.0044\right)\tau }-e^{\left(0.00083CL_{\text{cr}}+\;0.0044\right)t_{\inf}}\right]}$ .

For adjusted regimens (derived from CDM-3): $AUC_{24}=24C_{T\;-\;SS}^{O}\cdot \frac{D_{d}^{A}}{D_{d}^{O}}\cdot \frac{t_{\inf}}{\tau }\cdot \frac{{e^{\left(0.00083CL_{\text{cr}}+\;0.0044\right)}}^{\tau }-\;1}{e^{\left(0.00083CL_{\text{cr}}+\;0.0044\right)t_{\inf}}-\;1}$ .

^d^ Determined by the following model (derived from FDM) for both the original and adjusted regimens: *AUC*_24_ = daily dose/*CL*_van_.

0, invalid; 1, valid.

**FIGURE 3:**
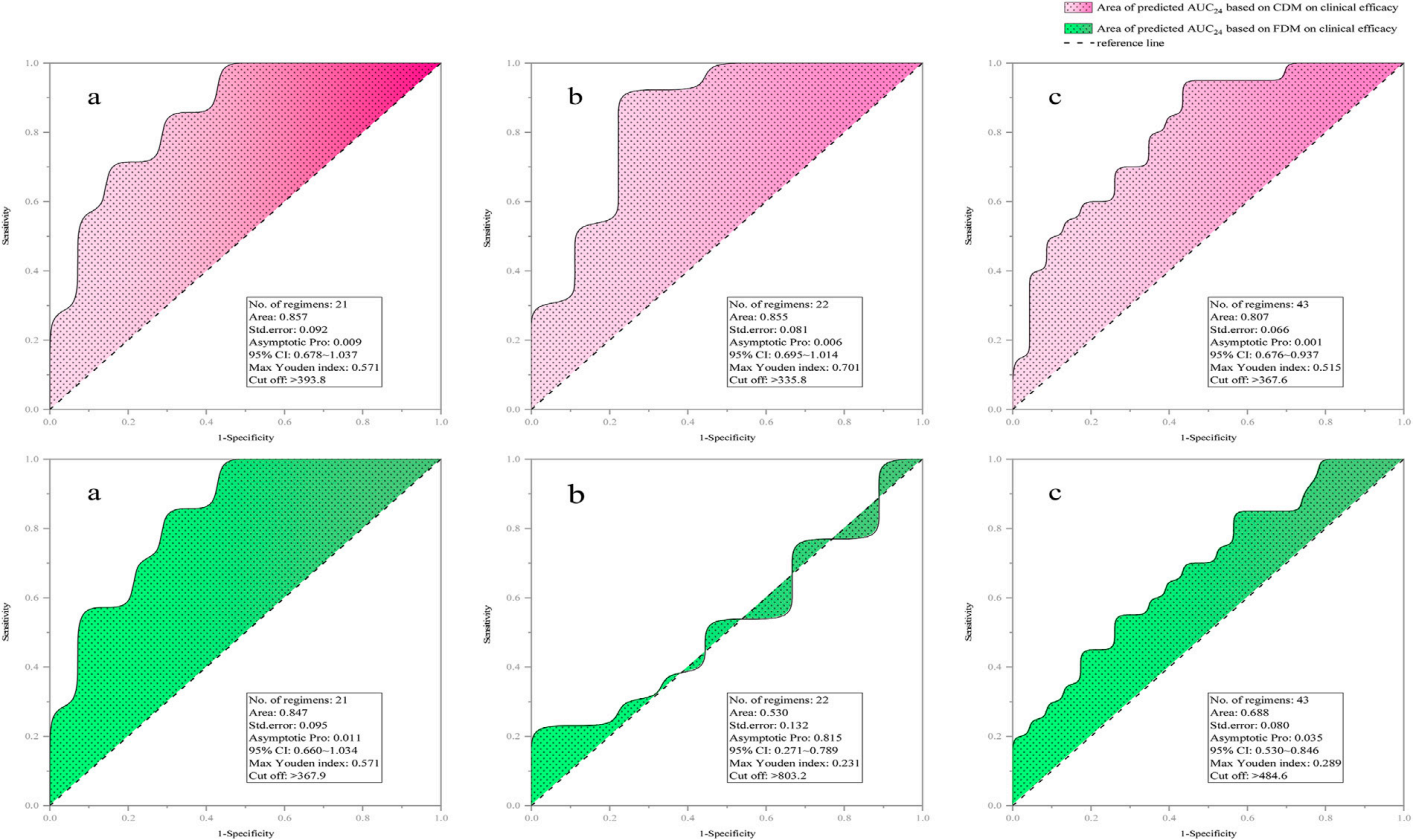
ROC curve of predicted AUC_24_ on clinical efficacy [**(a)** for original regimens (N = 21), **(b)** for adjusted regimens (N = 22), and **(c)** for all regimens (N = 43)]. AUC_24_, daily area under the concentration–time curve to minimal inhibitory concentration; CDM, customized dose models; FDM, frequently-used dose model; CI, confidence interval.

Figure 3 provides the ROC curve reflecting the predicted AUC_24_ on clinical efficacy. Compared with FDM, CDM exhibited a higher AUC_R_ that exceeds 0.8 (with a lower *p*-value between AUC_24_ and clinical efficacy), regardless of whether in original regimens [0.857 (0.009) v.s. 0.847 (0.011)], adjusted regimens [0.855 (0.006) v.s. 0.530 (0.815)], or total regimens [0.807 (0.001) v.s. 0.688 (0.035)]. Indicatively, CDM both in the original and adjusted regimens but FDM only in the original regimens was acceptable, and CDM-based AUC_24_ both in the original and adjusted regimens but FDM-based AUC_24_ only in the original regimens had a significant correlation with clinical efficacy. Compared with CDM, FDM showed an extremely high AUC_24_ cutoff (803.2 v.s. 335.8) in clinical efficacy prediction for adjusted regimens, suggesting that FDM may overestimate the required dose in order to achieve efficacy.

**FIGURE 4:**
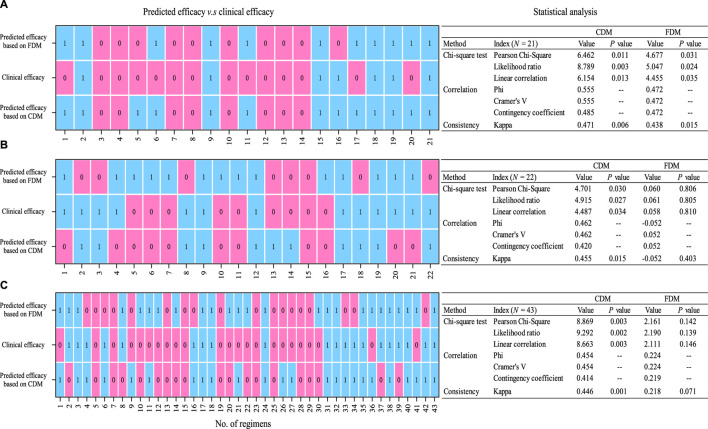
Statistical analysis (chi-squared test and correlation and consistency measurement) of predicted efficacy on clinical efficacy [**(A)** for original regimens (N = 21), **(B)** for adjusted regimens (N = 22), and **(C)** for all regimens (N = 43). 0, invalid; 1, valid]. CDM, customized dose models; FDM, frequently used dose model.

Figure 4 displayed the correlations and consistency of predicted efficacy on clinical efficacy. Compared with FDM, CDM showed higher Pearson chi-squared (with a reduced *p*-value), Kappa (with a reduced *p*-value), and Phi/Cramer’s V values, regardless of whether in the original regimens [Pearson chi-square: 6.462 (0.011) v.s. 4.677 (0.031), Kappa: 0.471 (0.011) v.s. 0.438 (0.015), and Phi/Cramer’s V: 0.555 v.s. 0.472], the adjusted regimens [Pearson chi-square: 4.701 (0.030) v.s. 0.060 (0.806), Kappa: 0.455 (0.015) vs. −0.052 (0.403), and Phi/Cramer’s V: 0.462 vs. −/+0.052], or the total regimens [Pearson chi-square: 8.869 (0.003) v.s. 2.161 (0.142), Kappa: 0.446 (0.001) v.s. 0.218 (0.071), and Phi/Cramer’s V: 0.454 v.s. 0.224]. Suggestively, FDM-based predicted efficacy only in the original regimen but CDM-based predicted efficacy both in the original and adjusted regimens showed a significant correlation and consistency with clinical efficacy.

### 3.3 Strategy formulation of individualized, dynamic, and full-course VAN dosing prediction from empirical to follow-up therapy

**FIGURE 5:**
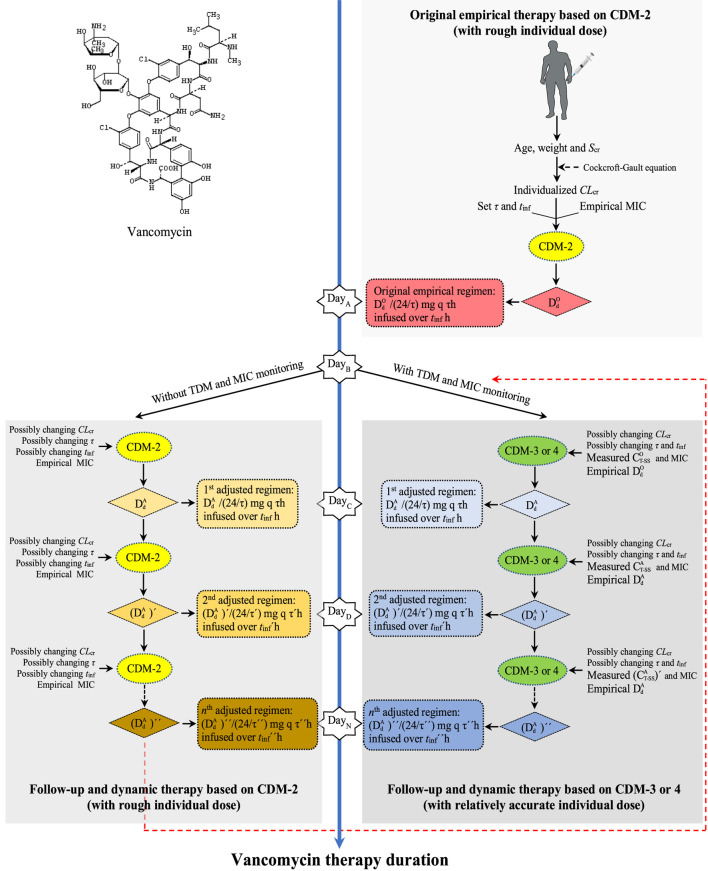
Strategy formulation of individualized, dynamic, and full-course VAN dosing prediction from empirical to follow-up therapy. CDM, customized dose model; Scr, serum creatinine; CLcr, creatinine clearance rate; τ, dosing interval; tinf, infusion time; MIC, minimal inhibitory concentration; $D_{d}^{O}$ , daily dose in the original delivery phase; $D_{d}^{A}$ , daily dose adjusted for the first time in the adjusted delivery phase; ${\left(D_{d}^{A}\right)}^{\prime }$ , daily dose adjusted for the second time in the adjusted delivery phase; ${\left(D_{d}^{A}\right)}^{\prime \prime }$ , daily dose adjusted for the *n*th time in the adjusted delivery phase.

CDM-based strategy formulation of individualized, dynamic, and full-course VAN dosing prediction from empirical to follow-up therapy is outlined in Figure 5. The estimation from the original dose based on CDM-2 to the follow-up and dynamic dose based on CDM-3 or CDM-4 fully illustrated the process of CDM-driven full-course prediction for customized and dynamic VAN dosing and the shift from imprecise empirical therapy to precise follow-up therapy.

### 3.4 Required $D_{d}^{O}$ and $D_{d}^{O}$ –CL_cr_ graphs for empirical VAN therapy

**FIGURE 6:**
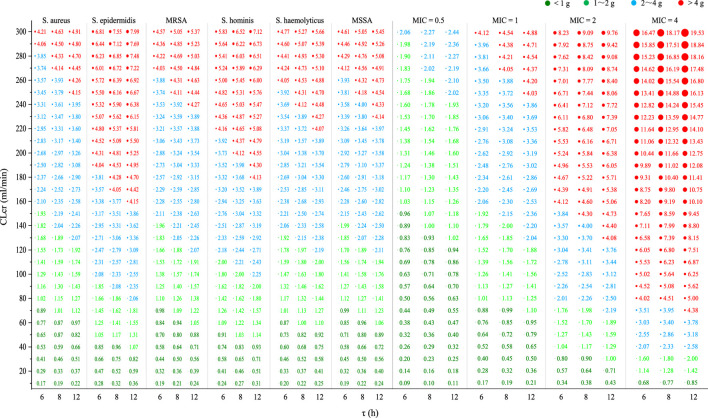
Required $D_{d}^{O}$ at various CL_cr_ under an AUC_24_/MIC of 400 and $D_{d}^{O}$ –CL_cr_ graphs for empirical VAN therapy. CL_cr_, creatinine clearance rate; τ, dosing interval; MIC, minimal inhibitory concentration; *S. aureus*, *Staphylococcus aureus*; *S. epidermidis*, *Staphylococcus* epidermidis; MRSA, methicillin-resistant *Staphylococcus aureus*; *S. haemolyticus*, *Staphylococcus* haemolyticus; *S. hominis*, *Staphylococcus* hominis; MSSA, methicillin-susceptible *Staphylococcus aureus*.

Figure 6 shows the required $D_{d}^{O}$ based on CDM-2 and $D_{d}^{O}$ –CL_cr_ graphs for *S. aureus*, *S. epidermidis*, MRSA, *S. haemolyticus*, *S. hominis*, and MSSA and for stains with a MIC of 0.5, 1, 2, and 4 mg/L at various CL_cr_ under an AUC_24_/MIC of 400. According to these graphs, the required $D_{d}^{O}$ and dosing regimen at different intervals [defined as $D_{d}^{O}$ /(24/τ) g q τh] for various populations can be easily determined. Notably, for the current *S. epidermidis* and strains with a MIC of 2 mg/L, patients with normal renal function (defined as with a CL_cr_ of 80–120 mL/min) should acquire a dosage of 3 g/d; while for strains with a MIC of 4 mg/L, these patients may acquire a dosage of 5 g/d or 6 g/d. However, against other target bacteria or strains, a standard dose of 2 g/d may be sufficient. Moreover, graphs indicated that at the same CL_cr_ and AUC_24_/MIC goal of 400, a shorter dosing interval can result in a lower $D_{d}^{O}$ .

## 4 Discussion

By mathematical modeling, this study successfully constructed CDM and dose–CL_cr_ graphs for VAN therapy. Our data supported the superiority and practicability of CDM in VAN dosing formulation and clinical efficacy prediction. CDM successfully informed and achieved individualized, dynamic, and full-course VAN dose prediction and dosing regimen formulation from original to follow-up therapy. Dose–CL_cr_ graphs instructed VAN dosing for various population and target bacteria in empirical therapy. Unlike BF, CDM-based VAN dose calculation can be performed using Microsoft^®^ Excel^®^ 2019 MSO (version 2112) or an application (e.g., Casio 991ES calculator) or a calculator (e.g., Casio fx-991ES) with advanced computing functions, making VAN dosing easier because it does not need professional software or technical support.

### 4.1 The CDM

Customized VAN dosage optimization has consistently been an interesting topic for discussion. When determining the required dose based on the target AUC_24_ or evaluating the AUC_24_ exposure based on the used dose, a simplified dose–AUC_24_ model (i.e., AUC_24_ = daily dose/CL_van_ or equivalently converted to daily dose = AUC_24_ × CL_van_) is widely utilized (Moise et al., 2000; Jeffres et al., 2006; Neuner et al., 2010; Kullar et al., 2011; Holmes et al., 2013; Lewis, 2018; Guo et al., 2019b). However, this simplified AUC_24_-based dose model might not be optimal for MII since (Song and Wu, 2022) 1) it does not adequately consider the effects of dosing parameters (including t_inf_ or v on AUC_24_ and τ on dose), which are very important, especially for adverse reactions of VAN (USP, 2018), and 2) the AUC_24_ here originates from i.v. bolus rather than from MII, and it actually measures the total AUC from 0 h to infinity (i.e., AUC_∞_) after a single dose but not the actual AUC_24_ from 0 h to 24 h (Rosenbaum, 2016; Drennan et al., 2019). Consequently, it could cause a significant departure from the expected dosage. Remarkably, this inference was just shown by an average AUC_24_ bias of 100.5 mg·h/L (Figure 2B) based on this model.

Some online VAN calculators, including ClinCalc (https://clincalc.com/Vancomycin/), VancoPK (https://www.vancopk.com/), and Vancovanco calculator (https://www.vancovanco.com/), enabled individualized VAN dosing. However, they utilized a trough-only AUC to estimate AUC and the above simplified dose–AUC_24_ model to calculate the VAN dose, which could cause a significant divergence from the expected dose. Reportedly, a considerable discrepancy was found between the AUC computed using the trapezoidal approach and the trough-only calculators (Keil et al., 2023), suggesting that the predicted dose based on these AUC results might be significantly biased. Other VAN calculators such as GlobalRPh (https://globalrph.com/medcalcs/vancomycin-dosing-bayesian-analysis/), Vancomycin dosing calculator (https://surgicalcriticalcare.net/vancomycin.html), JavaPK (https://www.pkpd168.com/jpkd), TDMx (https://tdmx.shinyapps.io/vancomycin/), Detroit Calculator (https://mad-id.org/vancomycin/implementation-resources/), and ID-ODS (https://motyocska.shinyapps.io/idods/) also provided computing services for individual VAN dosage. Despite Bayesian modeling being used, these calculators appear not to provide bases for dose calculation and not to have sufficient data to support their respective superiority and applicability in dose prediction. Observationally, the AUC calculated by ClinCalc, VancoPK, TDMx, and Detroit Calculator are not very accurate (Belz et al., 2023), indicating that the accuracy of the dose computed based on this AUC is, likewise, lowered. Additionally, not all calculators, like TDMx and GlobalRPh, are designed with ease of use in mind (Detroit Medical Center Vancomycin AUC Calculator, n.d.). Understandably, difficulty in selection posed by various calculators, the distinction and imprecision in the predicted results, and their relatively complicated online operation hinder the widespread use of these calculators and, thus, emphasize the importance of a workable and straightforward method for personalized VAN delivery.

To date, direct integration of PK/PD and TDM to create a simple predictive model to guide personalized VAN delivery has not received sufficient attention. Based on the dosing practice in which prescribers often adjust VAN dosage using existing experience, simplex trough target, and available TDM, this study simulated the concentration change and established CDM that integrates the PK/PD, physiological, dosing, and TDM factors by using the trapezoidal method. CDM not only directly guides the dosage calculation but also informs full-course prediction for VAN dosing from the initial to follow-up dose formulation. CDM-3 and CDM-4, in particular, provide a clear illustration of the conjugate of TDM with PK/PD for personalized VAN delivery. They also expose the strategic decision-making process for dosage adjustment based on TDM and the experiential/original dose, enabling patients to promptly reach the PK/PD target through timely dose adjustment. This instruction is very helpful when formulating dosage by adjusting the target value of the parameters to produce the desired effect in the following situations: 1) determining how much to increase the dosage by enhancing the setting of the PK/PD target when taking into account the low permeability of VAN in tissues like the lung or decreasing the dosage by decreasing the setting of the PK/PD target when considering the renal toxicity of VAN and 2) determining how to change dosage by altering the target setting of τ, t_inf_, and MIC when considering changes in dosing parameters and bacterial susceptibility. Furthermore, Microsoft^®^ Excel^®^ 2019 MSO (version 2112), an application (e.g., Casio 991ES calculator), or a calculator (e.g., Casio fx-991ES) with sophisticated computational features can handle the entire CDM-based dosage calculation process in one go, significantly streamlining laborious online operations and, thus, enhancing the scheme formulation experience.

In addition, CDM is a competitive individualized dose model. The present study used the Bland–Altman analysis of AUC_24_ to measure the consistency of CDM and FDM in predicting dosages, the AUC_R_ of the ROC curve to compare the performance of CDM and FDM in clinical efficacy predictions, and the Phi/Cramer’s V and Kappa values to evaluate the correlations and consistency of CDM- and FDM-based predicted efficacy on clinical efficacy. When a limit of ±48 mg·h/L for the AUC_24_ bias was used to evaluate the consistency and an AUC_R_ value of at least 0.8 was used to approve a dose model, data showed that 55.8% (24 of 43) of 43 regimens have inconsistent AUC_24_ and that in the performance evaluation of 43 regimens, the predicted AUC_24_ based on CDM has an AUC_R_ value of 0.807, but the predicted AUC_24_ based on FDM has an AUC_R_ value of only 0.688. It indicates that CDM and FDM are inconsistent in predicting dosages and that CDM is acceptable, whereas FDM is unacceptable in predicting AUC_24_. In addition, a Phi/Cramer’s V value of 0.454 for CDM *v.s.* that of 0.224 for FDM and a Kappa value of 0.446 for CDM *v.s.* that of 0.218 for FDM between the predicted and clinical efficacy imply that the predicted efficacy based on CDM has a better correlation and consistency with clinical efficacy compared with that based on FDM. These findings suggest that AUC_24_ based on CDM has better predictive performance for clinical efficacy, thus inversely indicating that the dosage based on CDM is more in line with individualized requirement, confirming the external validity of CDM in predicting dosages.

However, it should be noted that in the performance evaluation of the models, a direct comparison between the model-based dose and actual dose was not made, mainly because of the following reasons: 1) model-based dose is a theoretical dose determined according to AUC_24_/MIC of 400 and individualized PK parameters, and it is, therefore, an individualized dose suitable for individuals; meanwhile, the actual dose is a common dose approved by instructions and is, therefore, a general population dose suitable for most populations. Prior to the current viewpoint that the dosage of VAN should be determined based on an AUC_24_/MIC of 400, this legal general dose may not be established and approved based on an AUC_24_/MIC of 400. Therefore, the difference between the model-based dose and actual dose may be large, and therefore, no necessary correlation exists. 2) In the binary classification for the judgment of the predicted efficacy, there is a lack of quantifiable index that can judge the efficacy according to the dose. Considering that AUC_24_/MIC is a quantifiable consensus index for VAN efficacy judgment and dosage formulation, the present study converts CDM and FDM equivalently into an AUC_24_ model and uses the AUC_24_ comparison to demonstrate the accuracy and superiority of the model in reverse.

Based on the competitive CDM, the present study predicted the empirical VAN dosages for six *Staphylococci* and four strains. Against current *S. epidermidis* or strains with a MIC of 2 mg/L, data indicated that VAN 2 g/d and 4 g/d provided adequate PK/PD exposure in individuals with a CL_cr_ of up to 80 mL/min and 150 mL/min, respectively. It inferred that VAN 4 g/d can still be empirically utilized in patients with a CL_cr_ of up to 150 mL/min, even for the hyposensitive *S. epidermidis* or strains with a MIC of up to 2 mg/L. Contrary to this inference, however, prior investigations have revealed that when the MIC of strains is > 1 mg/L, the more sensitive daptomycin should be considered (Samura et al., 2022). For strains with a MIC of 4 mg/L, data showed that the general population (defined as those with normal renal function or a CL_cr_ of 80–120 mL/min) may acquire a dose of 5–6 g/d to reach adequate PK/PD exposure, suggesting that VAN may not be the best choice for this case, given its renal toxicity at a dose of >4 g/d (Lodise et al., 2008). However, against current *S. aureus*, MRSA, *S. haemolyticus*, *S. hominis*, and MSSA or strains with a MIC of ≤1 mg/L, standard 2 g/d offered sufficient PK/PD exposure and, thus, could have enough antibacterial competency in the general population. Reportedly, compared with MRSA strains with a lower MIC, VAN has been observed to have higher failure rates when confronted those with a MIC of ≥1.5 mg/L (van Hal et al., 2012). The present study confirmed this observation as Figure 6 showed that compared with a MIC of ≤1 mg/L, VAN, at the same dosage, dramatically reduced the intended population at a MIC of ≥2 mg/L when an AUC_24_/MIC goal of 400 was used as a measure of VAN efficacy. These findings provide us with preliminary reference for the empirical use of VAN in the face of these bacterial infections.

### 4.2 The limitations

The CDM is established based on the TDM, MIC, and AUC_24_/MIC target (especially CDM-3 and CDM-4). Therefore, the precision of these parameters determines the accuracy of the CDM-based dose. An accurate recording of drug dosing and sampling times, drug stability following sampling, establishment and feasibility of TDM methodology, measurements for total or free drug, and promptness in returning results affect the accuracy of the TDM report (Rawson et al., 2021a). Likewise, the methodology of MIC determination (e.g., use of automated systems, antimicrobial gradient strips, or broth microdilution), drawbacks of *in vitro* determination (e.g., failing to provide information on *in vivo* microbial characteristics), and other factors not considered in MIC determination (e.g., the site of infection, drug penetration, biofilm formation, and inoculum load) affect the accuracy of the MIC report (Rawson et al., 2021a; Dijkstra et al., 2021). Therefore, TDM and MIC reports derived from a laboratory usually fail to reflect the true scenarios. The AUC_24_/MIC target of 400 for VAN activity is largely derived from MRSA bloodstream infections (Rybak et al., 2020) and is an artificial consensus target. Therefore, with regard to whether it can be applied to other infections, no consensus has currently been reached. Moreover, we are unable to account for clinical decision-making with a hard cutoff of the target AUC_24_/MIC of 400 since the real-world decision-making based on individual variations is far more intricate than these predefined targets. Thus, these factors make it impossible for CDM to predict doses very accurately in practice, although it is a competitive model. Therefore, individual dosages for empirical VAN therapy based on CDM may need to be modified. In addition to these factors, these dosages are frequently impacted by presumptions on the distribution of individual parameters (e.g., CL_cr_ and t_inf_) since they are simulated.

Although the external validity demonstrates the superiority of CDM in dosage predictions, the limited sample size, theoretical features, and retrospective study design restrict the generalizability of CDM. It is, therefore, necessary to perform prospective research to further confirm the validity of CDM, which is also our next research plan. Nonetheless, CDM gives us preliminary recommendations on subsequent dose formulation. Moreover, some scholars may be worried about how adaptable the CDM will be in various patients with different physiopathology or PK changes. Since the CDM is developed based on the PK/PD theory and integrates the two parameters (i.e., CL_cr_ and C_T-SS_) that best reflect changes in physiopathology or PK and the concept of dynamic dosing is precisely proposed based on these changes, it is applicable to various patients and, therefore, exactly reflects its value.

## 5 Conclusion

To the best of our knowledge, this is the first study to use the trapezoidal approach for the prediction of individualized, dynamic, and full-course VAN dosing to build CDM that integrates the PK/PD, physiological, dosing, and TDM factors. Our data validate the superiority and predictability of CDM in VAN dose formulation. CDM simplifies the dose calculation process and provides instructions from initial to follow-up dose formulation. As current common methodologies, the single-point trough-based TDM technology and Bayesian modeling are still limited in individualized and dynamic VAN dosing, and CDM offers a straightforward and useful supplemental approach, especially in resource-constrained situations. By mathematical modeling of the VAN dosage, the goal of predicting individual doses is achieved, thus promoting “mathematical knowledge transfer and application” and providing reference for quantitative and personalized research on similar drugs.

## Data Availability

The original contributions presented in the study are included in the article/Supplementary material; further inquiries can be directed to the corresponding author.
